# Preschoolers and Adults Learn From Novel Metaphors

**DOI:** 10.1177/09567976231165267

**Published:** 2023-04-17

**Authors:** Rebecca Zhu, Alison Gopnik

**Affiliations:** Department of Psychology, University of California, Berkeley

**Keywords:** metaphor, relational reasoning, learning, concepts, cognitive development, open data, preregistered

## Abstract

Although adults use metaphors to guide their thinking and reasoning, less is known about whether metaphors might facilitate cognition earlier in development. Previous research shows that preschoolers understand metaphors, but less is known about whether preschoolers can learn from metaphors. The current preregistered experiment investigated whether adults (*n* = 64) and 3- and 4-year-olds (*n* = 128) can use metaphors to make new inferences. In a between-subjects design, participants heard information about novel artifacts, conveyed through either only positive metaphors (e.g., “Daxes are suns”) or positive and negative metaphors (e.g., “Daxes are suns. Daxes are not clouds.”). In both conditions, participants of all ages successfully formed metaphor-consistent inferences about abstract, functional features of the artifacts (e.g., that daxes light up rather than let out water). Moreover, participants frequently provided explanations appealing to the metaphors when justifying their responses. Consequently, metaphors may be a powerful learning mechanism from early childhood onward.

Metaphors are figurative utterances that directly compare concepts from one domain with concepts in other, unrelated domains. Metaphors occur frequently in both everyday language (e.g., “I got lost in a sea of people”) and famous creative works (e.g., Emily Dickinson’s “‘Hope’ is the thing with feathers”). For human adults, metaphors can facilitate communication and provide effective frameworks for reasoning about abstract concepts, thus influencing attention, memory, and information processing (Camp, 2009; Thibodeau et al., 2017). Moreover, metaphors are a force for creative change across many disparate domains: For example, metaphors facilitate the development of new insights about old concepts in art and poetry (Camp, 2009; Kulvicki, 2020), new word meanings in language (Bowdle & Gentner, 2005; Camp, 2006; Holyoak & Stamenkovíc, 2018), and new discoveries and theories in science (Kuhn, 1993; Nersessian, 2015).

Researchers across multiple domains of cognitive science have previously argued that metaphors are a powerful source of innovation and conceptual change in adults. For example, in the history of science, Kuhn (1993) noted the importance of the comparison between “atoms” and “solar systems” in the discovery of the structure of atoms. In psycholinguistics, Lakoff and Johnson (1980) argued that metaphors are fundamental to human cognition, shaping how we think and reason about the world around us. In philosophy, Camp (2006, 2009) suggested that metaphors frequently provide a new perspective on old information—in other words, a novel way of thinking about known concepts. Consequently, metaphors are a sophisticated cognitive mechanism that can facilitate learning, thinking, and reasoning in adults (Thibodeau et al., 2017) and, overall, are an important component of human innovation and creativity.

Although researchers have investigated the influence of metaphors on human adult cognition, less is known about whether and how metaphors might impact thinking and reasoning in children. Might metaphors, which facilitate cognitive innovation and creativity in adults, serve a similar function for young children? Researchers have posited that cognitive mechanisms for “learning by thinking,” such as analogy, explanation, and mental simulation (Lombrozo, 2019), may be critical for young children’s acquisition of new knowledge. Specifically, mechanisms for learning by thinking can help young children construct novel explanations, imagine alternative possibilities, and generate analogies. Consequently, these mechanisms may help expand young children’s conceptual repertoire by going beyond current empirical data to create new ideas and solutions (Xu, 2019). Given that metaphors and analogies provide new perspectives on known information (Camp, 2006, 2009) in a manner that might facilitate further discovery for adults, it is possible that metaphors might be an important mechanism for learning and conceptual change in childhood, too.

There is little research on metaphor as a learning mechanism in early childhood, in part because developmental psychologists previously believed that young children had serious difficulties understanding metaphors at all (Demorest et al., 1983; Silberstein et al., 1982; Winner, 1997; Winner et al., 1980). However, more recent research argued that metaphor comprehension emerged earlier in ontogenesis (Pouscoulous & Tomasello, 2020; Zhu et al., 2020). Indeed, recent work showed that children develop sophisticated relational-reasoning abilities in their preschool years (Christie & Gentner, 2014; Goddu et al., 2020; Hochmann et al., 2017; Kroupin & Carey, 2022) or even earlier (Anderson et al., 2018; Walker et al., 2016; Walker & Gopnik, 2017). Moreover, additional research demonstrated that preschoolers understand other kinds of nonliteral language, such as metonyms (Falkum et al., 2017; Köder & Falkum, 2020; Zhu, 2021) and metaphors (Pouscoulous & Tomasello, 2020; Zhu et al., 2020, in press). For example, 3-year-olds already understand metaphors based on perceptual similarities (e.g., “The bottle with the big belly” to refer to a round bottle over a slender bottle; Pouscoulous & Tomasello, 2020) but not abstract-motion/space relations (e.g., “Time flies by”; Özçaliskan, 2005). In contrast, 4-year-olds successfully understand metaphors based on abstract similarities, such as shared functions (e.g., “Roofs are hats”; Zhu et al., 2020, in press) or motion/space relations (e.g., “Ideas pass through one’s mind”; Özçaliskan, 2005, 2007). Although this recent research on preschoolers’ metaphor comprehension is promising, it is unknown whether preschoolers can use metaphors to learn. Empirical research shows that preschoolers can represent similarities between two familiar concepts—for example, by noticing that sponges and clouds both hold water (Gentner, 1988; Zhu et al., 2020)—but it is still unknown whether preschoolers can also use metaphors to make entirely new inferences—for example, by using their knowledge of clouds to learn about the features of novel artifacts. If preschoolers can make new inferences from metaphors, this may suggest that metaphors are a powerful learning mechanism early in ontogenesis.

**Statement of Relevance**

Adults frequently use metaphors, not only to communicate with others but also to guide their thinking and reasoning, to draw new conclusions, and to create new concepts. However, less is known about whether young children can also understand and learn from metaphors. In the current experiment, 3-year-olds, 4-year-olds, and adults heard metaphors about novel toys (e.g., “Daxes are suns”) and used these metaphors to infer previously unknown information about the toys’ functions (e.g., that daxes light up). Moreover, when asked to justify their responses on the task, both adults and children frequently provided explanations appealing to the metaphors (e.g., “Because daxes are suns, and suns light up”). Overall, this research suggests that young children possess an early-emerging capacity to understand and use complex nonliteral language. Moreover, metaphors may be a powerful cognitive tool that helps both adults and young children learn about the world around them.

Thus, the present study is the first to investigate whether preschoolers can use metaphors to generate new information and ideas. Specifically, we investigated whether 3-year-olds, 4-year-olds, and adults can make inferences from novel metaphors. To ensure that participants interpreted the utterances as nonliteral metaphors comparing two conceptually distinct items (e.g., “Juliet is the sun”) rather than literal category statements (e.g., “Juliet is a girl”), we specified that all the novel items were artifact kinds (i.e., toys) and compared these novel items with natural or social kinds (e.g., animals, occupations). Given preschoolers’ knowledge of the distinctions between natural and artifact kinds (e.g., Gelman, 1988), they should recognize the nonliteral character of these statements. Moreover, the present experiment used relatively concrete concepts (e.g., animals, toy) that are easy to depict and familiar to young children. In a between-subjects design, we tested 3-year-olds, 4-year-olds, and adults in one of two experimental conditions. In the positive-only condition, we presented participants with positive metaphors (e.g., “Feps are ballerinas”), similar to the ones they might hear in naturalistic contexts (i.e., metaphors are often presented alone rather than contrasted with other metaphors). In the positive-and-negative condition, we presented participants with both positive and negative metaphors (e.g., “Feps are ballerinas. Feps are not soldiers”) to rule out lower-level associations (e.g., hearing “ballerina” might encourage participants to select the ballerina-related response without attending to and reasoning about both concepts involved in the metaphor).

Given previous research showing that 4-year-olds understand sophisticated, abstract metaphors (Özçaliskan, 2005, 2007; Zhu et al., 2020, in press) but that 3-year-olds succeed at metaphor-comprehension tasks only sometimes (Özçaliskan, 2005; Pouscoulous & Tomasello, 2020), we hypothesized that 4-year-olds, but not 3-year-olds, would succeed on the present metaphor-learning task. Surprisingly, we found that participants in all age groups and conditions—including 3-year-olds—accurately formed metaphor-consistent inferences about the novel artifacts. Consequently, these results suggest that preschoolers as young as 3 years old both understand and learn from metaphors.

## Open Practices Statement

Deidentified data, analysis scripts, and preregistrations have been made publicly available via OSF and can be accessed at https://osf.io/wzsx6. In the initial preregistration, we stated that only 4-year-olds would be included in the positive-and-negative condition. However, given 4-year-olds’ success in this initial condition, we added a second preregistration with an additional condition (i.e., positive only) and age group (i.e., 3-year-olds). Because the second preregistration stated that we would analyze data from both age groups and conditions together, we report all data as a single experiment. The materials used in this study are available on request.

## Method

### Participants

During recruitment, we adhered to a preregistered stopping rule of 32 participants in each age group and condition. This sample size provided approximately 80% power to detect a moderate to large effect size (*d*s = 0.5–0.7) and is consistent with or larger than sample sizes used in previous experiments on children’s metaphor comprehension (e.g., Zhu et al., 2020, in press). Consequently, 64 adults (*M* = 21.09 years, *SD* = 1.38 years, range = 18.20–25.64 years; 44 females, 18 males, two nonbinary), 64 four-year-olds (*M* = 4.51 years, *SD* = 0.30 years, range = 4.02–4.99 years; 30 females, 34 males), and 64 three-year-olds (*M* = 3.70 years, *SD* = 0.22 years, range = 3.13–3.98 years, 32 females, 32 males) participated in the study. All participants predominantly spoke and heard English (i.e., more than 50% of the time). We tested five additional 4-year-olds but excluded their data because of experimenter error (*n* = 3), external interference (*n* = 1), or refusal to complete the experiment (*n* = 1), as well as four additional 3-year-olds, whose data we excluded because of experimenter error (*n* = 3) or refusal to complete the experiment (*n* = 1). Children were recruited from a database; adults were recruited from a university campus and reflected local convenience samples. Adult participants were Asian (55%), White (22%), Latina (17%), mixed White-Asian (5%), or mixed Latina-Asian (1%). Child participants were White (50%), mixed White-Asian (17%), Asian (15%), Latina (10%), Black (3%), mixed White-Black (1%), mixed White-Middle Eastern (1%), mixed Black-Latina (1%), mixed Asian-Latina (1%), and mixed White-Asian-Black (1%). All experiments reported in this article were approved by the Committee for the Protection of Human Subjects at a U.S. university. All adult participants and parents of child participants provided informed consent.

### Stimuli and procedure

The experimenter presented participants with stories online over Zoom, which participants viewed using either a computer or a tablet. The experimenter introduced the paradigm by showing participants a picture of a girl and saying, “This is my friend Sophie. Sophie makes a lot of toys in her toy factory. She’s going to tell you about her toy, and then your job is to guess what Sophie’s toy can do! Ready to play?”

### Positive-only condition

**Fig. 1.**
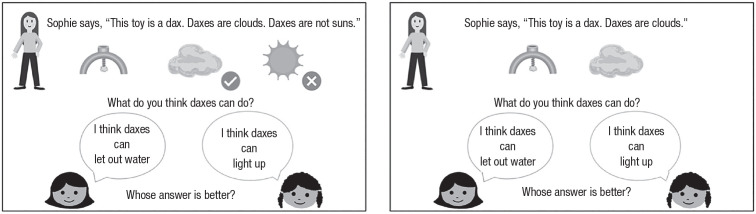
Example of a test trial in the positive-and-negative condition (left) and the positive-only condition (right).

In the positive-only condition, participants were presented with only positive metaphors (e.g., “Daxes are clouds”). On each trial, the experimenter introduced a novel toy using a positive metaphor (e.g., “Sophie says, ‘This toy is a dax. Daxes are clouds’”). As the experimenter presented this information verbally, clip-art pictures (e.g., a novel toy and a cloud) appeared on the screen. Then, the experimenter asked about the toy’s function (e.g., “What do you think daxes can do?”). A person appeared on the left side of the screen and provided an answer consistent with one of the metaphors (e.g., “This person says, ‘I think daxes can let out water,’” an inference consistent with the cloud metaphor). Then, another person appeared on the right side of the screen and provided an answer consistent with another metaphor (e.g., “This person says, ‘I think daxes can light up,’” an inference consistent with the sun metaphor). The experimenter then asked the participant to choose between the two answers (i.e., “Whose answer do you think is better?”). For an example trial, see Figure 1. Once the participant answered by providing a response (e.g., “let out water”), the experimenter began the next trial. No feedback was provided. On the final trial, the experimenter asked participants for an explanation (e.g., if the participant selected “let out water” on the final trial, the experimenter followed up by asking, “Why do you think daxes let out water?”).

Each participant received eight trials. For a list of metaphors and corresponding inferences for each novel toy that participants were shown, see Table 1. Each trial’s structure followed the design described above, in which a participant had to infer the function of the novel toy using the metaphor presented. Trial order was randomized. Within participants, we counterbalanced the left/right placement of the correct answer. Across participants, we counterbalanced which metaphor was presented, such that half the participants heard one positive metaphor (e.g., “Daxes are clouds”), and the other half heard another positive metaphor (e.g., “Daxes are suns”). Moreover, across participants, we counterbalanced which of the eight metaphors participants encountered on the final trial.

**Table 1.** Metaphors and Corresponding Inferences for Each Novel Toy

Novel toy	Metaphor A	Metaphor B	Inference A (corresponding to metaphor A)	Inference B (corresponding to metaphor B)
Daxes	Daxes are clouds.	Daxes are suns.	Daxes can let out water.	Daxes can light up.
Lubbos	Lubbos are snails.	Lubbos are bees.	Lubbos can move slowly.	Lubbos can buzz loudly.
Wugs	Wugs are songbirds.	Wugs are cheetahs.	Wugs can make music.	Wugs can move quickly.
Feps	Feps are ballerinas.	Feps are soldiers.	Feps can twirl around.	Feps can shoot pebbles.
Biboos	Biboos are seagulls.	Biboos are kangaroos.	Biboos can fly.	Biboos can bounce.
Blickets	Blickets are eyes.	Blickets are teeth.	Blickets can help you see things.	Blickets can help you chop things.
Meelees	Meelees are stars.	Meelees are ponds.	Meelees can sparkle.	Meelees can hold water.
Pims	Pims are ducks.	Pims are fireflies.	Pims can float in the water.	Pims can glow in the dark.

### Positive-and-negative condition

The positive-and-negative condition was similar to the positive-only condition, except that participants heard both positive and negative metaphors (e.g., “Daxes are clouds. Daxes are not suns.”). This ensured that participants’ correct responses were driven by a sensitivity to the overall metaphor rather than by lower-level associations (e.g., hearing “cloud” might encourage participants to select the cloud-related response without attending to the full metaphor). Pictures were accompanied with either a small green checkmark to indicate a positive metaphor (e.g., a checkmark placed beside the cloud reminded participants that daxes are clouds) or a small red “x” to indicate a negative metaphor (e.g., an “x” placed beside the sun reminded participants that daxes are not suns).

In addition to the counterbalancing in the positive-only condition, the positive-and-negative condition also counterbalanced which metaphors were positive and negative across participants, such that half of the participants heard that daxes were clouds and not suns, and the other half of the participants heard that daxes were suns and not clouds. Moreover, across participants, we counterbalanced whether the positive or negative metaphors were mentioned first, such that half of the participants heard the positive metaphor first (e.g., “Daxes are suns. Daxes are not clouds”), and the other half of the participants heard the negative metaphor first (e.g., “Daxes are not suns. Daxes are clouds”).

## Results

### Main analyses: proportion of correct responses

In the following analyses, the dependent variable was the proportion of correct, metaphor-consistent responses. In a preregistered analysis involving preschoolers, a between-subjects analysis of variance (ANOVA) with age group (3-year-olds, 4-year-olds) and condition (positive only, positive and negative) yielded a main effect of age group—the proportion of correct responses increased with age, *F*(1, 124) = 9.22, *p* = .003—and a marginal effect of condition—the proportion of correct responses increased slightly in the positive-and-negative condition, *F*(1, 124) = 3.18, *p* = .08. There was no interaction between age group and condition, *F*(1, 124) = 0.07, *p* = .79.

In an additional exploratory analysis involving both preschool and adult samples, a between-subjects ANOVA with age group (3-year-olds, 4-year-olds, adults) and condition (positive only, positive and negative) yielded a main effect of age group—the proportion of correct responses increased with age, *F*(2, 186) = 33.08, *p* < .001—and a main effect of condition—the proportion of correct responses increased in the positive-and-negative condition, *F*(1, 186) = 6.05, *p* = .01. There was no interaction between age group and condition, *F*(2, 186) = 0.06, *p* = .94. We examined differences across conditions by age group: Adults performed better in the positive-and-negative condition than the positive-only condition, *t*(62) = 2.74, *p* = .008. However, there was no performance difference between the two conditions for 4-year-olds, *t*(62) = 1.67, *p* = .10, and 3-year-olds, *t*(62) = 0.96, *p* = .34.

**Fig. 2.**
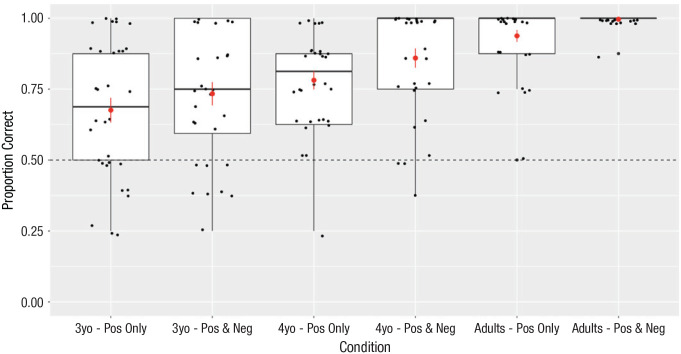
Proportion of correct responses by age group (3-year-olds, 4-year-olds, adults) and condition (positive only, positive and negative). The vertical length of each box indicates the interquartile range, and the horizontal line represents the median. The red dot is the mean. Error bars show 1 *SD* and whiskers show the entire range of responses. Dots indicate individual data.

In preregistered analyses, we found that 4-year-olds also selected the correct response at significantly above-chance levels in both the positive-only condition, *M* = 78.13%, 95% confidence interval (CI) = [71.55, 84.70], *t*(31) = 8.72, *p* < .001, and the positive-and-negative condition, *M* = 85.94%, 95% CI = [79.00, 92.88], *t*(31) = 10.56, *p* < .001. In exploratory analyses, we found that participants in all age groups and conditions selected the metaphor-consistent response at significantly above-chance levels (see Fig. 2). Adults overwhelmingly selected the correct response in both the positive-only condition, *M* = 93.75%, 95% CI = [89.47, 98.03], *t*(31) = 8.72, *p* < .001, and the positive-and-negative condition, *M* = 99.61%, 95% CI = [98.81, 100.41], *t*(31) = 127, *p* < .001. Three-year-olds also selected the correct response at significantly above-chance levels in both the positive-only condition, *M* = 67.58%, 95% CI = [58.65, 76.51], *t*(31) = 4.01, *p* < .001, and the positive-and-negative condition, *M* = 73.36%, 95% CI = [64.98, 81.74], *t*(31) = 5.69, *p* < .001. Because all age groups and conditions performed at significantly above-chance levels (all *p*s < .001) and there was no difference between conditions for preschoolers (i.e., the primary population of interest), we aggregated data across the two conditions for the remainder of the analyses.

We found that participants of all ages selected the metaphor-consistent response at above-chance levels. In a preregistered analysis, 4-year-olds’ performance was also above chance, *M* = 82.03%, 95% CI = [77.28, 86.78], *t*(63) = 13.48, *d* = 1.68, *p* < .001. In an exploratory analysis, adults’ performance was above chance, *M* = 96.68%, 95% CI = [94.44, 98.92], *t*(63) = 41.61, *d* = 5.20, *p* < .001, and 3-year-olds’ performance was also above chance, *M* = 70.47%, 95% CI = [64.47, 76.46], *t*(63) = 6.82, *d* = 0.85, *p* < .001.

### Additional analyses: by trial

In additional exploratory analyses, we found that a significant proportion of adults and children performed above chance levels by responding correctly on 100% of trials (binomial test, *p* < .01). Specifically, 84.38% of adults (54/64), 39.06% of 4-year-olds (25/64), and 26.56% of 3-year-olds (17/64) responded correctly on all eight trials. All three proportions are significantly higher than one would expect by chance (binomial test, all *p*s < .001).

We also conducted exploratory analyses examining participants’ performance on individual trial types. Adults selected the correct response at above-chance levels on all eight trials (*p* < .001 on all trials): Responses ranged from 92.19% correct (on the ballerina/soldier trial) to 100% correct (on the songbird/cheetah trial). Similarly, 4-year-olds selected the correct response at above-chance levels on all eight trials (*p* < .001 on all trials): Responses ranged from 78.13% correct (on the snail/bee trial) to 85.94% correct (on the seagull/kangaroo trial). Three-year-olds selected the correct response at above-chance levels on seven of eight trials (*p <* .02), but responses were at chance on the cloud/sun trial (*p* = .21). Three-year-olds’ responses ranged from 57.81% correct (on the cloud/sun trial) to 79.69% correct (on the snail/bee and seagull/kangaroo trials). Significant results remained significant after correction for multiple comparisons (Benjamini & Hochberg, 1995).

### Explanations

In exploratory analyses, we examined participants’ explanations. Each participant provided a single explanation on the final trial, leading to a total of 192 explanations. Explanations were coded without knowledge of participants’ performance. Explanations were coded in four categories: explicit metaphor, implicit metaphor, toy, and irrelevant. Explicit-metaphor explanations appealed explicitly to the natural/social kind in the positive metaphor (e.g., “Blickets are eyes and eyes are used to see things,” “It’s a seagull”). Implicit-metaphor explanations appealed to the features of the natural/social kind involved in the positive metaphor but did not name the natural/social kind (e.g., “They have wings,” “To catch their prey”). Toy explanations appealed to features of the novel toys (e.g., “They have a little bucket at the end,” “They have batteries”). Irrelevant explanations were nonsensical or nonresponses (e.g., “It sounds like the right answer,” “I don’t know”). Two coders coded all explanations. Intercoder reliability was 93%, converging on the same category for 178 out of 192 explanations.

The majority of adults and 4-year-olds, and approximately half of 3-year-olds, appealed to the metaphors in their explanations. Most (93.75%, 60/64) of adults appealed to metaphors: Specifically, 87.5% (56/64) provided explicit-metaphor explanations, 6.25% (4/64) provided implicit-metaphor explanations, 4.69% (3/64) provided toy explanations, and 1.56% (1/64) provided irrelevant explanations. Similarly, 68.75% (44/64) of 4-year-olds appealed to metaphors: Specifically, 45.31% (29/64) provided explicit-metaphor explanations, 23.44% (15/64) provided implicit-metaphor explanations, 7.81% (5/64) provided toy explanations, and 23.44% (15/64) provided irrelevant explanations. Nearly half (48.44%, 31/64) of 3-year-olds appealed to metaphors: Specifically, 29.69% (19/64) provided explicit-metaphor explanations, 18.75% (12/64) provided implicit-metaphor explanations, 3.12% (2/64) provided toy explanations, and 48.44% (31/64) provided irrelevant explanations.

We conducted exploratory analyses examining participants’ performance on the basis of whether their explanations involved metaphors (i.e., explicit-metaphor and implicit-metaphor explanations) or not (i.e., toy and irrelevant explanations). Adults performed at above-chance levels, whether they appealed to metaphors, *M* = 97.08%, 95% CI = [94.83, 99.37], *t*(59) = 41.81, *p* < .001, or not, *M* = 90.63%, 95% CI = [71.58, 109.67], *t*(3) = 6.79, *p* = .007. There was no difference between adults who appealed to metaphors and adults who did not, *t*(62) = 1.40, *p* = .17. Four-year-olds also performed at above-chance levels, whether they appealed to metaphors, *M* = 86.93%, 95% CI = [82.16, 91.71], *t*(43) = 15.60, *p* < .001, or not, *M* = 71.25%, 95% CI = [61.19, 81.31], *t*(19) = 4.42, *p* < .001. However, 4-year-olds who appealed to metaphors performed significantly better than 4-year-olds who did not, *t*(62) = 3.29, *p* = .002. Similarly, 3-year-olds also performed at above-chance levels, whether they had appealed to metaphors, *M* = 78.23%, 95% CI = [70.03, 86.43], *t*(30) = 7.03, *p* < .001, or not, *M* = 63.18%, 95% CI = [54.84, 71.52], *t*(32) = 3.22, *p* = .003. However, 3-year-olds who appealed to metaphors performed significantly better than 3-year-olds who did not, *t*(62) = 2.62, *p* = .011. All results remained significant after correction for multiple comparisons (Benjamini & Hochberg, 1995). Overall, both 3-year-olds and 4-year-olds who provided explanations appealing to metaphors performed better than preschoolers who did not.

## General Discussion

This work shows that preschoolers can not only understand metaphors but also use metaphors in the service of further thinking and reasoning. Specifically, preschoolers can use metaphors to learn new information about novel artifacts. Three-year-olds, 4-year-olds, and adults successfully formed metaphor-consistent inferences, both when given only positive metaphors (e.g., “Blickets are teeth”) and when given positive and negative metaphors (e.g., “Blickets are teeth. Blickets are not eyes”). Participants’ success in the positive-and-negative condition suggests that they were indeed using the metaphors, rather than lower-level associative strategies, to guide their responses. Moreover, we found that preschoolers still performed exceptionally well on both conditions of this metaphor-learning task, even under extremely stringent criteria. Specifically, in all age groups, a significant proportion of participants performed at above-chance levels by responding correctly on all eight trials. Adults and 4-year-olds also responded at significantly above-chance levels on all eight individual trials, whereas 3-year-olds responded at significantly above-chance levels on seven of the eight individual trials.

Our preregistration noted the exploratory nature of any explanation analyses, given that children often struggle to generate sensible explanations. However, we found that the majority of adults and 4-year-olds, as well as approximately half of 3-year-olds, possessed the impressive ability to appeal to the metaphors when providing an explanation for their responses. Moreover, 3- and 4-year-olds who appealed to metaphors in their explanations performed significantly more accurately than 3- and 4-year-olds who did not.

Despite previous research suggesting that 3-year-olds possess a limited or inconsistent ability to reason about abstract relations (Hochmann et al., 2017) and understand abstract metaphors’ relations (Özçaliskan, 2005), we found that 3-year-olds performed at significantly above-chance levels in the current metaphor-learning task. Moreover, almost half of 3-year-olds generated explanations that appealed to metaphors. Consequently, 3-year-olds demonstrated a sophisticated ability to form additional inferences from novel metaphors. Our data also revealed an age effect: Specifically, although both 3-year-olds and 4-year-olds selected metaphor-consistent inferences, 4-year-olds were significantly more accurate. This age effect suggests that children’s metaphor comprehension may still be developing in the preschool years. Indeed, there are multiple possibilities for what cognitive abilities might be developing, and these possibilities are not mutually exclusive. For example, previous research shows that metaphor comprehension relies in part on conceptual knowledge (Keil, 1986), relational-reasoning capacities (Gentner, 1988; Zhu et al., 2020), and executive function capacities (Carriedo et al., 2016), all of which are still developing in the preschool years.

Although the present research demonstrates that even preschoolers are able to learn from metaphors, there are still many fruitful directions for future research. For example, given the surprising success of 3-year-olds in the current task, it is possible that even younger children might be able to learn from metaphors, metaphorical thinking, and abstract relations and that this might contribute to their development of new knowledge and concepts. Moreover, although the current experiment used metaphors involving abstract similarities between *concrete* concepts, future research might explore whether preschoolers can also understand and learn from metaphors involving abstract similarities for *abstract* concepts. Indeed, one possible account of why 3-year-olds successfully understood and made inferences from metaphors in the current experiment, but not in previous research, is that the former involved only concrete concepts, whereas the latter involved more abstract concepts (e.g., “time flies by,” “ideas pass through one’s mind”; Özçaliskan, 2005). This is consistent with previous research suggesting that children have more difficulty understanding metaphors involving abstract mental states (e.g., “The prison guard is a hard rock”) than metaphors involving shared perceptual similarities (e.g., “Her perfume was bright sunshine”; Winner et al., 1976). Given that conceptual knowledge is important for children’s metaphor comprehension (Keil, 1986), one possibility is that preschoolers are more familiar with, and thus have an easier time reasoning about, concrete concepts than abstract concepts. To understand and learn from a metaphor, children must be confident in their knowledge of the source domain and have an appropriate context to extend it to the target—for example, it would be hard to take advantage of a metaphor comparing the atom with the solar system if one did not already possess some understanding of both concepts. In the current study, we used simple physical processes (e.g., clouds produce rain, ballerinas dance) that preschoolers were familiar with and applied them to a problem that children regularly encounter—namely, inferring the functions of artifacts. This contrasts with earlier studies using more abstract concepts, such as mental states or time (Özçaliskan, 2005; Winner et al., 1976), that 3-year-olds were less likely to understand. However, more work is required to conclusively reconcile these findings and understand the scope of preschoolers’ abilities to comprehend and use metaphors.

One limitation of the present research is that it relied on U.S. convenience samples, which are predominantly White, urban, and upper middle class. Given evidence of early diversity in children’s relational-reasoning abilities across contexts (e.g., Carstensen et al., 2019), these findings may not generalize to children growing up in other early environments. Future research should investigate when children across various cultures and contexts begin to understand and learn from metaphors.

Overall, the present experiment contributes to a growing body of literature suggesting that preschoolers may possess a relatively sophisticated ability to understand and use nonliteral language (Falkum et al., 2017; Pouscoulous & Tomasello, 2020; Zhu, 2021; Zhu et al., 2020). Moreover, by demonstrating that preschoolers can make additional inferences from novel metaphors, the present research suggests that metaphors may be a powerful learning mechanism that could allow children to acquire new information. Just as metaphors have facilitated novel scientific discoveries in the history of science (Kuhn, 1993) and higher-order cognitive processes in human adults (Thibodeau et al., 2017), metaphors may also contribute to conceptual innovation and learning in early childhood. Interestingly, because metaphors frequently provide a new perspective (Camp, 2006, 2009) without necessarily providing new information, they may be a powerful case of “learning by thinking,” allowing for the acquisition of new knowledge and concepts with little or no additional data (Lombrozo, 2019; Xu, 2019). Metaphors and metaphorical thinking, then, may contribute to preschoolers’ remarkable learning abilities.

## Transparency

*Action Editor:* Sachiko Kinoshita

*Editor:* Patricia J. Bauer

*Author Contributions*

**Rebecca Zhu:** Conceptualization; Data curation; Formal analysis; Funding acquisition; Investigation; Methodology; Project administration; Writing – original draft; Writing – review & editing.

**Alison Gopnik:** Conceptualization; Investigation; Methodology; Project administration; Supervision; Writing – review & editing.
